# Nutritional Status of Non-Institutionalized Adults Aged over 65. Study of Weight and Health in Older Adults (PYSMA)

**DOI:** 10.3390/nu13051561

**Published:** 2021-05-06

**Authors:** Felipe Mozo-Alonso, José P. Novalbos-Ruiz, Juan C. Duran-Alonso, Amelia Rodríguez-Martin

**Affiliations:** 1 Colegio Oficial Farmacéuticos de Cádiz, Calle Isabel la Católica, 22. Cádiz, 11004 Cádiz, Spain; felipemozo@farmaciasanfrancisco.com; 2 School of Medicine, Cádiz University, Plaza Falla 9, 11003 Cádiz, Spain; 3 Hospital Juan Grande, Glorieta Félix Rdguez, de la Fuente, Jerez de la Frontera, 11408 Cádiz, Spain; juaduralo@gmail.com; 4 Nursing School of Cádiz University, Avda. Ana de Viya 52, 11009 Cádiz, Spain; amelia.rodriguez@uca.es

**Keywords:** elderly, nutritional status, obesity, overweight, polypharmacy, physical activity

## Abstract

Background. A significant increase in the prevalence of malnourishment, obesity, and sarcopenic obesity has been observed in developed countries over the last few decades. In Spain, this especially happens in populations over 65 who are not institutionalized. Differences in lifestyle, medication, and economic capacity partially explain this increase. Objective. To study the nutritional status of a population of 65 year-olds and subjects who are not institutionalized, in the Cádiz region (Spain). Methods. Observational, transversal study carried out on 2621 subjects who are 65 years old and over, with a direct weight and height measurement, in 150 pharmacy offices from 44 locations. A mobile application was designed for homogeneous data collection in all the pharmacy offices. The data required from all subjects was gender, age, postal code, social security contribution regime, if the patient lives alone, type of food consumed as the main meals, level of physical activity, polypharmacy, weight, and height. Results. The prevalence of overweight and obesity amounts to 82.2% of the population (43.2% overweight and 39% obese). We found an inverse relationship between the prevalence of overweight and obesity with carrying out physical activity and having full dinners. Conclusion. We identify the need to reinforce the messages to the elderly aimed at maintaining adequate physical activity and assessing the quality and quantity of dinners, as well as reducing, as much as possible, the treatments that may lead to weight gain.

## 1. Introduction

The elderly comprise the fastest-growing population group, and the WHO projecting a total of 1.2 billion people over the age of 60 years by 2025 [1].

A working group on nutrition for the Spanish Society of Geriatrics and Gerontology (SEGG) published a consensus document [2] assessing the nutritional status of the elderly. The consensus’ objective was to improve the diagnosis of nutritional disorders that are common among this age group, which are sometimes underdiagnosed [3]. Structured scales are available to help with nutritional screening; the Mini Nutritional Assessment (MNA) is the most endorsed for diagnosing malnutrition in the elderly population, but it is not used to diagnose obesity [4,5]. 

Physical examinations of the elderly must include anthropometric evaluations: such as height, weight, and BMI. Several studies have shown that the prognostic value of BMI in the elderly is different from that of younger adults due to changes in body composition [6]. The loss of muscle mass is reflected in the BMI, with the consequent decrease in the prevalence of overweight and obesity in the elderly population or in those with sarcopenia, leading to an increase in morbidity and mortality (U-shaped pattern between BMI and risk of morbidity and mortality [6,7]. A BMI between 25 and 28 kg/m^2^ is associated with better health.

The Longitudinal Aging Study Amsterdam (LASA) [8], conducted in a population-based sample in the Netherlands, showed that the agreement between objective and self-perceived body weight status in older Dutch adults was low (Kappa < 0.2). Only 4.4% of obese men and 12.3% of obese women perceived their body weight status correctly; so a higher age (in women), lower educational level, and higher BMI (all) are all associated with greater underestimation of body weight status. An improved body weight perception in the elderly is necessary to increase the impact of public health campaigns, especially in patients with multiple pathologies.

Depending on the consensus document used, there are different cut-off points for the BMI measurement. The SEGG proposes the following BMI cut-off values: 30 kg/m^2^ for obesity; 27–29.9 for overweight; 22–26.9 for normal weight; 18.5–21.9 for underweight, and <18.5 kg/m^2^ for malnutrition [2]. On the other hand, The Spanish Society for the Study of Obesity (SEEDO) considered BMI values >25 for overweight and >30 for obesity, making no distinction between younger adults and the elderly [9]. In our study, we opted for this second choice (SEEDO), due to it being the most widely used in previous published studies. During the last two decades, there has been a significant increase in the prevalence of obesity among the over 65s, especially among those who do not reside in long term care institutions, in the older age brackets, with the lowest socioeconomic levels [10,11]. The term “sarcopenic obesity” came into use in the late 1990s to describe people who had excess body fat (obesity) along with a significant loss of muscle mass (sarcopenia) [12,13,14]. In 2018, the EWGSOP2 [12] consensus used the loss of muscle strength as the main defining factor of sarcopenia, in addition to the decrease in quantity and quality of muscle mass. In Spain, the prevalence of sarcopenic obesity affects 15% of non-institutionalized older adults [15], with an increase according to age, reaching 20% in those older than 80 years [16,17,18]. A multicenter study carried out in Spain (EXERNET) found that the prevalence of sarcopenic obesity affected 15% of non-institutionalized older adults [15]. This increases according to age, representing 20% in the over 80s [16,17,18]. On the other hand, it is necessary to emphasize the importance of the Mediterranean Diet (DM) as a model of a quality diet, associated with a reduction in mortality and with an improvement in the quality of life in older people, supported by a 30% decrease in processes cardiovascular disease and an improvement in cognitive aspects, as reflected in the PREDIMET study [19].

Different factors could contribute to nutritional modifications in the elderly, such as educational level [20], previous diseases, or pharmacological treatments received [21,22].

One of the most common problems that we face in ageing societies is our sedentary lifestyle. We can improve this by encouraging physical exercise along with adequate nutritional advice to ensure correct caloric intake, the appropriate intake of different types of nutrients [23], and their proper distribution in main meals [24,25,26].

With regards to sedentarism, a recent study suggests that moderate exercise alone or in combination with high-protein dietary supplements may provide benefits to body composition, as well as functional tests (grip strength and gait speed) in subjects with sarcopenic obesity living in the community [23,26]. What should be noted is not only the importance of physical exercise acting on sedentary lifestyle in older people, but that it must also be carried out regularly and continuously [27]. Among social risk factors in the elderly, loneliness and isolation stand out. Usually, older people living alone spend less time preparing food than those who live with their partner or family. The full-time presence of a caregiver means that the older person is more likely to eat proper meals. Yet, when the caregiver is only present with the older person for a few hours in the morning, breakfast and lunch are usually better than dinner [23]; another social risk factor to be considered is the reduced purchasing power for many of the oldest adults [21,26,28].

Older adults with good household assets and access to medical services were less likely to experience multimorbidity of chronic non-communicable diseases, whereas obese and centrally obese older adults, older adults who were current smokers and current drinkers, and those with a family history of chronic non-communicable diseases had a greater probability of multimorbidity [23].

A modification in the composition of the diet has been observed in older people with sarcopenia. This fact requires the special attention of duly trained health professionals and the provision of meals that comply with dietary recommendations especially, the supply of protein [21,24]. With all this background in mind, the aim of our study was to examine the nutritional status of the non-institutionalized 65 years old and over population who live in the southwest area of Spain (Cadiz province). The data of users from the age of 65 and over who went to the different pharmacies in the province of Cádiz were collected.

## 2. Materials and Methods

### 2.1. Subjects and Study Design

We have conducted an epidemiological, observational and cross-sectional study, without a control group, on adults aged 65 or over, taking direct measurements for height and weight at 150 pharmacies in 44 locations in the province of Cádiz between June and July 2018.

As inclusion criteria, we included those individuals who were 65 years old or over who went to the pharmacy and who agreed to participate in the study. As exclusion criteria, we only consider the inability to answer, but those who had specific diets or dietary patterns were not excluded. Likewise, the criteria of complete main meals do not imply that patients with specific dietary patterns are considered incomplete.

To calculate sample size, we have considered the entire population aged 65 or over in the province (270,133 inhabitants, according to the 2017 Spanish census*) and the expected prevalence of overweight (50%). We assumed a 95% power ((1-beta = 0.95) and (1-alpha/2 = 0.95)) for a 2-sided *p*-value of 0.05 and a design effect of 2, obtaining a minimum sample size of 1440 subjects. The study involved 30.2% of the province’s pharmacy offices (150 of 497), located in 44 of the 45 municipalities in the province. The participation of the greatest number of municipalities was prioritized to ensure the representativeness of rural populations, so in the selection of pharmacies, the population size (rural, semi-urban, and urban municipalities) and their location (coastal or inland municipalities) were considered too.

### 2.2. Experimental Procedure

To recruit patients, a marketing campaign was carried out prior to the study in pharmacies using posters, pamphlets, and by displaying the study’s image on existing screens. After the end of the pharmaceutical care, the objectives of the study were explained to the elderly, and their voluntary participation was requested. Appropriate consent and authorization were obtained prior to participation to state that they were willing to participate voluntarily and that we could use their data anonymously. The acceptance rate was higher than 95%. The study protocol was approved by the Ethics Committee of our official Institution.

A mobile application was designed to homogenize data collection from all pharmacies. We anonymously recorded the following data: gender, age, postcode, social security contribution category, if the individual lives alone, food groups consumed in main meals, the level of physical activity, and if they were taking five medications or more (polypharmacy), as well as the weight and height of the patient (obtained whilst standing without shoes and with light clothing (no coats or jackets).

The anthropometric measurements were carried out in the pharmacies by the pharmacists using calibrated scales and measuring rods (RD 244/2016, Law 32/2014). Although the materials for the anthropometric measures (scales, measuring rods, and tape measures) were not uniform (exact models), all scales and measuring instruments for public use in pharmacies are calibrated and verified at least once a year.

As a socioeconomic indicator, we collected the patient’s economic contribution scheme to pharmacy expenses according to their recognized income (exempt from payment contributions of 10%, 30% or 60%).

During the time of the study, the patient’s medication history was available electronically, subject to previous authorization, as was access to their social security electronic card. If the patient was taking 5 medications or more, such as psychotropic drugs (antidepressants, anxiolytics, antipsychotics, opioids, antiepileptics) and some drugs for cognitive impairment (anticholinergics, NMDA antagonists, and others), then the information was recorded.

According to the SENC Healthy Nutrition Guide [29], the best way to achieve adequate nutritional status is to incorporate a wide variety of foods into our daily diet, according to the different groups of the nutritional pyramid. To evaluate nutritional habits, a questionnaire, prepared ad-hoc by the researchers (adapted from the SENC Guide), was used, considering the frequency of the daily intake of the basic nutritional groups. The survey included: daily intakes in the main meals (breakfast, lunch, dinner) and registering the consumption of dairy products and derivatives: proteins (meat, fish, eggs, legumes), carbohydrates (bread, cereals, pasta), vegetables, and fruits. A main meal was considered complete when at least three different basic food groups were consumed.

Physical activity was collected based on the information provided by the users through the questionnaire adapted by the researchers [30]. Thus, the level of physical activity was classified, considering 3 parameters: duration, frequency (number of times per week), and intensity. Information was collected on household chores, leisure time, and tasks in the previous 7 days. Physical activity status was considered as follows: sedentary when the subject does not leave their home or does so occasionally; light activity if they carry out activities related to daily life (such as personal care, cooking, or shopping) and leaving the house regularly; moderate activity if they carry out some physical leisure activity or sport one to three days a week; and intense activity if this activity is taken up more than three days a week. We use BMI classification criteria adopted by the SEEDO [9] as an exploratory parameter of nutritional assessment, such as their cut-off points. In the study protocol, referral to the family doctor was advised for patients with BMI <18.5 (malnutrition) or >35 (extreme obesity).

### 2.3. Statistical Analysis

The association between physical activity, nutritional characteristics, residence in rural/semi-urban/urban municipalities, and polypharmacy versus the frequency of overweight and obesity was evaluated using the Chi-squared proportions comparison test and Fisher’s exact test. As a multivariate analysis, we used logistic regression. Prior to the comparison of mean values, we checked the normality of the data with the Kolmogorov–Smirnov test and performed Levene’s test of equality of variances. T-test, ANOVA, and Mann–Whitney U were used to analyze the existence of significant differences in BMI figures by economic level, physical activity, variety of main meals, polypharmacy, and consumption of psychotropic drugs, and for multiple pairwise comparisons we used Tukey’s method. We performed the statistical analysis with the SPSSv24 program, and the level of statistical significance used was *p* < 0.05.

## 3. Results

A total of 2621 adults aged 65 or over participated from the province of Cádiz; 38.2% of them were male and 61.8% were female. The mean age of male and female was 74.81 years (SD 6.61) and 73.89 years (SD 6.35), respectively. Seventy-five percent of this group resided in municipalities with populations with more than 20,000 inhabitants (semi-urban), and only 8.4% lived in rural areas (<5000 inhabitants). With regards to financial income, 9% of the participants did not have any recognized income (TSI001) and 79% received less than 18,000 euros (TSI002A).

Table 1 shows the distribution by age group and gender, as well as some of the features of their dietary habits, physical activity, sedentary lifestyle, if they live alone, medication use, and their income category. We found statistically significant differences (*p* < 0.05) between gender in physical activity (greater in men), consumption of psychotropic drugs (greater in women), prevalence of overweight (men), and obesity (women).

With regards to dietary habits, and according to the study definition [26], only 10.2% of men and 12.7% of women had a complete breakfast, and 43.8% of men and 42.1% of women had a complete dinner. We did not find any association related to whether the older adult lived alone or not.

Most of the 65 years old and over population does light physical activity or has a sedentary lifestyle. 52.3% of men and 57.3% of women carry out daily living activities and go for regular strolls; 18.4% have a sedentary lifestyle (20.2% women compared to 15.4% men). Roughly a quarter (26.3%) of the elderly do some type of physical activity in their leisure time, with more men doing this than women (32.4% vs. 22.4%).

Table 2 shows the BMI percentiles according to gender and age to show the nutritional status assessment for these age groups. As shown in Table 2, there is a decrease in body mass index (BMI) in all percentiles with the aging of the population. This decrease is greater in overweight and obese subjects (especially in women). Likewise, the value of the median BMI (28.35 in men and 29.02 in women) shows that in both populations there is a high prevalence of excess weight.

The prevalence of overweight and obesity (Table 3) equates to 82.2% of the population (43.2% overweight and 39% obese); in the male population, the prevalence of overweight is higher (49.1%) while in women there is a predominance of obesity (42.3%). The average BMI among those aged over 65 corresponds to that of an overweight and obese population, with higher values among women than men (29.2–29.7 vs. 28.4–28.9, respectively).

The values of overweight are similar in all age groups, while obesity decreases significantly as age increases. In the 65–69 age bracket, it affects 41.2% of the population, while only 27.4% of the over 85s group is affected. There is a significant drop in average BMI measurements correlating to an increasing age.

With regards to the size of the living areas, there is a greater prevalence of obesity in rural areas, where it represents up to 47.2%. In urban and semi-urban areas, the prevalence of obesity is lower, but they are very similar to each other (38.9% and 38%, respectively).

There is an inverse relationship between the prevalence of overweight and obesity on one hand and physical activity and eating complete dinners on the other. The prevalence of obesity is much higher for sedentary subjects (53%), affecting men and women to the same degree. As physical activity increases among older adults, the prevalence of obesity goes down and the prevalence of overweight and normoweight goes up.

In the group who have complete dinners, 19.3% are normoweight and 37.6% obese. The percentage of obese is higher among those who do not have complete dinners (15.6% normoweight and 41.8% obese).

If we analyze the existence of differences between rural and urban settings for the parameters already mentioned, we find that, regarding physical activity, in rural areas, fewer older adults have a sedentary lifestyle, which is lower than in semi-urban areas and urban ones (13.4% vs. 23.8% and 17.8%, respectively), and more perform moderate to intense physical activity (31.5% vs. 26.7% and 26.3%, respectively). Dietary habits for the elderly are very similar in the three settings considered (Table 4).

We have not found a significant difference with regards to having a complete breakfast, lunch, or dinner in the three location types. However, we do find an increase in medication use among the rural population (63% take five medications or more compared to 55.8% in semi-urban areas and 54.4% in urban areas), largely relating to antidepressants and anxiolytics. Antidepressant use is notably higher in rural areas than in semi-urban or urban areas (35.3% compared to 27.9% and 25.3%, respectively). Regarding financial income level, those who do not have any recognized income and those who receive less than 18,000 euros are more likely to take five medications or more (61.3% and 56.4%, respectively, compared to 23.8% of those who receive more than 100,000 euros) (Table 4).

Psychotropic drugs are included among the different pharmacological groups most consumed in the elderly population. Within this group, antidepressants and anxiolytics are the most consumed. There is a high frequency of consumption in women (23.2% antidepressants and 39.5% anxiolytics) that almost doubles the frequency of consumption in men (11.6% antidepressants and 23% anxiolytics). Twelve percent of people over 65 consume, concurrently, antidepressants and anxiolytics, a percentage that reaches 17% in rural areas.

**Figure 1:**
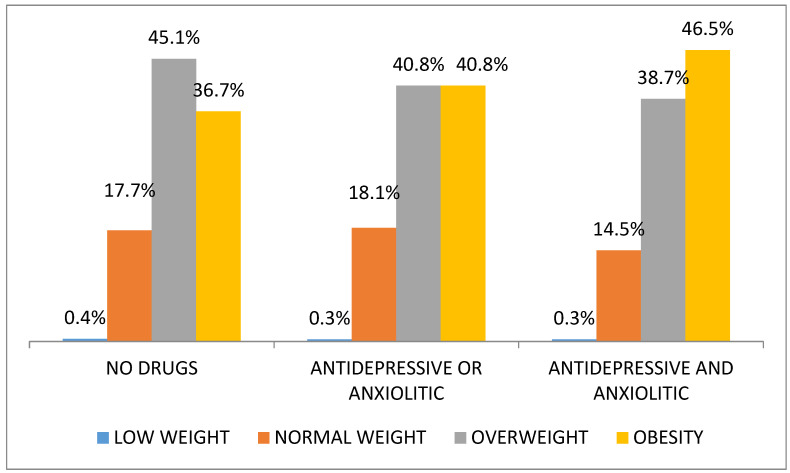
Nutritional status according to antidepressants or anxiolytic drugs use.

When analyzing the relationship between BMI and the consumption of the most prescribed psychotropic drugs in this population segment, we find that the average BMI values are higher (*p* = 0.02) in the subjects who simultaneously consume antidepressants and anxiolytics (BMI 29.7 95% CI 29.2–30.3) in comparison to the population who do not consume them (BMI 28.4 95% CI 28.6–29.1). As shown in Figure 1, the consumption of these drugs is associated with a higher prevalence of obesity.

The multivariate study has revealed that the variables associated to a greater probability of having a BMI over 25 Kg/m^2^ in the over 65s are the following: a sedentary lifestyle (OR = 1.60 95% CI 1.01 2.55), eating an incomplete dinner (OR = 1.28 95% CI 1.03 1.58), and taking five or more medications (OR = 1.33 95% CI 1.06 1.65). Age has a significant influence and has a greater effect on the 65–74 age bracket (OR = 2.16 95%CI 1.43 3.25) (Table 5). The financial contribution rate was negatively associated with overweight/obesity (OR = 0.83 95% CI 0.71 0.98); the higher the financial contribution, the lower the risk. The location type does not appear to influence the prevalence of overweight and obesity.

## 4. Discussion

The BMI values for the over 65s in our country are alarming. More than 80% of this group are overweight or obese, and these figures are rising. In the Exernet study [20], carried out during the period 2008–2010 among 2438 non-institutionalized subjects aged over 65, the percentage of men found to be overweight was 51.7% and obese was 30.6%. In women, the figures were 41.7% and 38.3%, respectively. In our study, carried out among 2621 participants, we found a 3% increase in obesity among men and 4% among women, directly reducing the percentage of overweight people.

The fall in mean BMI values with increasing age reflects, in addition to the loss of muscle mass, a survival bias due to higher levels of mortality among those older than 70 years [28]. When we look at the prevalence of overweight and obesity in relation to age, obesity figures increase as people age from 65 to 80 years old, at the same time as overweight figures decrease. From the age of 80 years old, the prevalence of obesity decreases according to age, and the prevalence of overweight increases [9]. On the one hand, this could reflect the relationship between BMI and survival due to the increased risk of mortality associated with obesity at these ages [22]. Additionally, we must considered that there are authors who have described up to 15% sarcopenia among this age group in Spain [15,31] as changes to body composition are produced in the ageing process, which can lead to underestimating the nutritional status of this population group [16]. Ghallaguer et al. [32] proposed that, for those older than 79 years, obesity should be assessed at 30% and 42% of overall body fat (in men and women, respectively) rather than using BMI values.

The frequency of full breakfasts that include dairy, cereals, and fruit among older adults is remarkably low. Less than 12% of this population meets the nutritional recommendations for breakfast. For our population group, in addition to a low frequency of breakfasts, almost 60% of the group do not have adequate dinners. We additionally found that not having a proper dinner had a more significant effect on the likelihood of being overweight and obese than inadequate breakfasts. We therefore find, at least in our setting, that we must help our oldest adults reassess the importance of having dinner.

Regarding physical activity among the elderly, 26.3% reported moderate or intense physical activity. This figure may seem too high. It has been considered due to the age in the age of the population, moderate or intense physical activity refers to doing physical activities in their leisure time one to three times a week or more than three times, regardless of the duration. Furthermore, we have to bear in mind that those examined in this study all frequented pharmacies. Therefore, we are overestimating intense physical activity (7.3%) and underestimating figures that represent the sedentary lifestyle (18.4%). Despite these figures, we did find a significant association between overweight/obesity and doing physical activity, as described in similar studies [33]. Physical activity is a key indicator of impaired functionality at these ages; it is related to the development of sarcopenia and osteoporosis and increased fat mass [16,17]. Furthermore, a sedentary lifestyle also produces an increase in fat mass [16]. All these factors should be taken into consideration when evaluating the apparent drop in obesity as age increases.

Overweight and obesity have been commonly associated with behaviors and habits considered normal in urban settings. However, the PREV-Ictus study, published in 2005 [34], already confirmed a higher prevalence of obesity in Spaniards over 65 in rural and semi-urban areas due to a more sedentary lifestyle. The towns in our study with fewer than 5000 inhabitants also showed higher levels of overweight and obesity. However, the multivariate analysis indicated that it is not the size of the population living in an area that determines the probability of overweight and obesity, but rather the physical activity of the person, their dietary habits (more specifically if they have adequate dinners), if they take five or more medications, and their income level (measured by their allowances). An important aspect to consider in this age range is polypharmacy. A significant association is observed between taking more than five medications and the prevalence of overweight and obesity. Studies show the correlation between the intake of anxiolytics and certain antidepressants with weight gain [35]. The comorbidity that requires the patient to take this medication can also predetermine overweight and obesity related to the sedentary lifestyle or deterioration of the patient.

It is a descriptive study, so causality could not be established. The present study has some limitations: only data for users who went to the pharmacy were collected; even though the instruments were all calibrated, they were not homogeneous in all pharmacy offices [36]; and the physical activity may have been overestimated. On the other hand, its strengths include the high number of subjects collected and the representativeness of pharmacy offices throughout the province of Cádiz.

Given the breadth of the sample and the representativeness of both rural and urban pharmacy offices throughout the province, the methodology can be applied to other areas, although different results could be obtained depending on the nutritional or physical activity characteristics of the users served

## 5. Conclusions

In conclusion, we need to act by targeting the older population if we want to reduce the prevalence of obesity, it being such an important risk factor for them. This can be done, acting on the three most important identified factors: physical activity, nutrition, and polypharmacy, by encouraging them to continue with recommended physical activity and removing the popular myth in our Mediterranean culture that dinners can consist of a light snack, thus giving them the importance they deserve. Furthermore, we should consider the potential influence that different treatments can have on weight gain in this segment of the population.

## Figures and Tables

**Table 1:** General characteristics of non-institutionalized aged 65s or over: Economic contribution pharmacy expenses, dietary habits, physical activity or a sedentary lifestyle, polypharmacy. PYSMA Study.

		Men		Women		
		*N*	%	*N*	%	*p*-Value
		1000	38.2%	1621	61.8%	
Age Group (years)	65–69	251	25.1%	486	30.0%	0.006 *
	70–74	261	26.1%	447	27.6%	
	75–79	217	21.7%	340	21.0%	
	80–84	186	18.6%	247	15.2%	
	≥85	85	8.5%	101	6.2%	
Economic Allowance—Economic Contribution Pharmacy Expenses (Euros-%)	TSI01 < 18.000€—0%	68	7.0%	154	9.9%	0.011 *
	TSI02 < 18.000€—10%	763	78.7%	1239	79.3%	
	TSI02B 18–100.000€—10%	71	7.3%	85	5.4%	
	TSI06B Work Insurance—30%	56	5.8%	76	4.9%	
	TSI05 > 100.000€—60%	12	1.2%	9	0.6%	
Variety Food Main Meals	Full breakfast	92	10.2%	189	12.7%	0.166 *
	Complete lunch	733	79.0%	1231	81.4%	0.104 *
	Complete dinner	403	43.8%	630	42.1%	0.421 **
Physical Activity	Sedentary lifestyle	149	15.4%	316	20.2%	0.000 *
	Light (walk)	507	52.3%	896	57.3%	
	Moderate (1–3 days/week)	213	22.0%	267	17.1%	
	Intense (>3 days/week)	101	10.4%	84	5.4%	
Polypharmacy and Drug Consumption Frequency	5 or more drugs	531	54.7%	868	55.5%	0.712 **
	Anxiolytics	223	23.0%	617	39.5%	0.000 **
	Antidepressants	113	11.6%	363	23.2%	0.000 **
	Anxiolytics+antidepressants	68	6.8%	242	14.9%	0.000 *
	Antiparkinsonians	45	4.6%	141	9.0%	0.000 **

* *p* value Chi-square test; ** *p* value Fisher’s exact test.

**Table 2:** BMI percentiles according to age group and gender.

Percentiles	Men (*n* = 1000)				
	65–69 years	70–74 years	75–79 years	80–84 years	≥85 years
P5	22.83	21.99	23.01	22.56	21.79
P10	23.97	24.11	23.81	24.08	22.79
P25	26.05	26.01	25.70	25.76	25.03
P50	28.87	28.31	28.04	28.28	28.27
P75	31.93	31.12	30.10	30.85	31.07
P90	34.77	33.45	33.23	33.41	33.68
P95	37.05	35.26	34.99	34.97	35.47
	**Women (*n* = 1621)**				
	**65–69 years**	**70–74 years**	**75–79 years**	**80–84 years**	**≥85 years**
P5	21.57	22.31	22.05	22.27	19.48
P10	22.83	23.93	23.48	23.71	21.83
P25	25.80	26.28	26.05	25.63	25.14
P50	29.02	29.38	29.77	28.93	28.03
P75	32.58	32.64	32.38	32.31	30.19
P90	36.69	36.29	35.47	35.20	34.52
P95	38.82	39.26	37.86	37.20	35.81

Background color indicate BMI < 25 in green or BMI > 30 in red.

**Table 3:** BMI distribution according to age group and gender.

		BMI			
	Age Group	<18.5	18.5–24.9	25–29.9	≥30
		Underweight	Normalweight	Overweight	Obesity
MEN	65–69		16.7%	40.2%	43.0%
	70–74	0.8%	13.8%	52.5%	33.0%
	75–79	0.5%	18.4%	55.8%	25.3%
	80–84	0.5%	16.1%	49.5%	33.9%
	≥85		24.7%	47.1%	28.2%
	All groups	0.4%	16.9%	49.1%	33.6%
WOMEN	65–69	0.2%	18.9%	40.5%	40.3%
	70–74	0.4%	14.5%	40.7%	44.3%
	75–79	0.3%	16.5%	35.3%	47.9%
	80–84		21.1%	38.1%	40.9%
	≥85	2.0%	22.8%	48.5%	26.7%
	All groups	0.4%	17.8%	39.6%	42.3%

**Table 4:** Level of physical activity, variety of food groups in main meals, and polypharmacy in adults aged 65 years old and over in rural, semi-urban, and urban areas. PYSMA Study.

	Types of Cities			
	Rural	Semi-Urban	Urban	Total
Level of physical activity				
Sedentary lifestyle	13.4% *	23.80%	17.80%	18.50%
Light physical activity (walk)	55.10%	53.50%	55.90%	55.40%
Moderate physical activity (1–3 days/week)	21.8% *	16.70%	19.00%	18.80%
Intense physical activity (>3 days/week)	9.70%	6.00%	7.30%	7.30%
Variety of food groups main meals				
Full breakfast	8.00%	11.40%	12.20%	11.70%
Complete lunch	79.20%	78.80%	81.10%	80.60%
Complete dinner	45.00%	44.20%	42.10%	42.70%
Polypharmacy	63.0% ^φ^	55.80%	54.40%	55.40%
(5 or more drugs)				

* T-Kendall 2.45 *p* = 0.014; ^φ^ Fisher’s exact test *p* = 0.022.

**Table 5:** Factors associated with the presence of overweight and obesity among non-institutionalized adults aged 65 years old and over. Multivariate analysis.

					95% C.I. EXP(B)	
	B	Standard Error	*p*-Value.	Exp(B)	Low	High
SEX	0.114	0.114	0.316	1.121	0.897	1.400
AGE GROUPS			0.001			
65–69 years	0.719	0.206	0.000	2.053	1.372	3.071
70–74 years	0.846	0.208	0.000	2.331	1.550	3.506
75–79 years	0.544	0.210	0.009	1.722	1.142	2.597
80–84 years	0.457	0.217	0.035	1.579	1.032	2.416
ECONOMIC CONTRIBUTION PHARMACY EXPENSES (TS)	−0.175	0.080	0.028	0.839	0.717	0.982
PHYSICAL ACTIVITY LEVEL			0.026			
Sedentary lifestyle	0.475	0.236	0.044	1.608	1.012	2.553
Light physical activity (walk)	0.321	0.201	0.109	1.379	0.931	2.043
Moderate physical activity (1–3 days/week)	0.007	0.217	0.974	1.007	0.659	1.540
FOOD VARIETY IN DINNER	0.248	0.109	0.023	1.281	1.034	1.586
POLIPHARMACY	0.285	0.112	0.011	1.330	1.068	1.657
Constant	1.005	0.324	0.002	2.733		

## Data Availability

The datasets used and/or analyzed during the current study are available from the corresponding author on reasonable request. Statistical analysis was performed with SPSS version 21. XI National Congress of Pharmaceutical Care (Cadiz. October 2019), 40th Congress of the Andalusian Society of Geriatrics and Gerontology (Jerez de la Frontera, Cádiz. October 2019), and II Jornada de Alimentación del Colegio Oficial de Farmacéuticos de Cádiz (May 2019).
